# Indirect Export of Reducing Equivalents From the Chloroplast to Resupply NADP for C_3_ Photosynthesis—Growing Importance for Stromal NAD(H)?

**DOI:** 10.3389/fpls.2021.719003

**Published:** 2021-10-20

**Authors:** Moritz Krämer, Hans-Henning Kunz

**Affiliations:** Department I, Plant Biochemistry and Physiology, Ludwig-Maximilians-University Munich, Munich, Germany

**Keywords:** C_3_ photosynthesis, redox, chloroplast, coenzymes, acclimation

## Abstract

Plant productivity greatly relies on a flawless concerted function of the two photosystems (PS) in the chloroplast thylakoid membrane. While damage to PSII can be rapidly resolved, PSI repair is complex and time-consuming. A major threat to PSI integrity is acceptor side limitation e.g., through a lack of stromal NADP ready to accept electrons from PSI. This situation can occur when oscillations in growth light and temperature result in a drop of CO_2_ fixation and concomitant NADPH consumption. Plants have evolved a plethora of pathways at the thylakoid membrane but also in the chloroplast stroma to avoid acceptor side limitation. For instance, reduced ferredoxin can be recycled in cyclic electron flow or reducing equivalents can be indirectly exported from the organelle via the malate valve, a coordinated effort of stromal malate dehydrogenases and envelope membrane transporters. For a long time, the NADP(H) was assumed to be the only nicotinamide adenine dinucleotide coenzyme to participate in diurnal chloroplast metabolism and the export of reductants via this route. However, over the last years several independent studies have indicated an underappreciated role for NAD(H) in illuminated leaf plastids. In part, it explains the existence of the light-independent NAD-specific malate dehydrogenase in the stroma. We review the history of the malate valve and discuss the potential role of stromal NAD(H) for the plant survival under adverse growth conditions as well as the option to utilize the stromal NAD(H) pool to mitigate PSI damage.

## Introduction

Oxygenic photosynthesis started to evolve over 3 billion years ago (Blankenship, 2010; Sánchez-Baracaldo and Cardona, 2020). It allowed for CO_2_ fixation into carbohydrates and other energy rich compounds releasing O_2_ as a byproduct. This enabled oxygen-requiring metabolic and biosynthetic pathways which form the basis for higher multicellular life on earth (Schirrmeister et al., 2013). Although the conversion of light energy into chemical energy has fascinated scientists for centuries, many aspects of the underlying biochemistry are still not fully understood. Land plants represent the main food resource for humans, livestock, and wild animals. In addition, their importance as renewable energy carriers and sustainable building materials have increased drastically over the last decades. In a world undergoing climate change with more adverse weather conditions, humankind has to overcome many challenges to meet the food and energy demand of the growing global population (Popp et al., 2014). As part of this effort, a detailed understanding of the molecular mechanisms governing photosynthetic efficiency in the plant chloroplast is indispensable. This includes knowledge on how plants rapidly acclimate to the dynamic growth conditions they face in the field e.g., shifts in light intensity and temperature. Several molecular processes housed in the chloroplast have emerged as critical for acclimation, emphasizing the central importance of the organelle (Kleine et al., 2021). Insights from this research will inform the design of crop plants with improved tolerance against adverse growth conditions.

**Figure 1:**
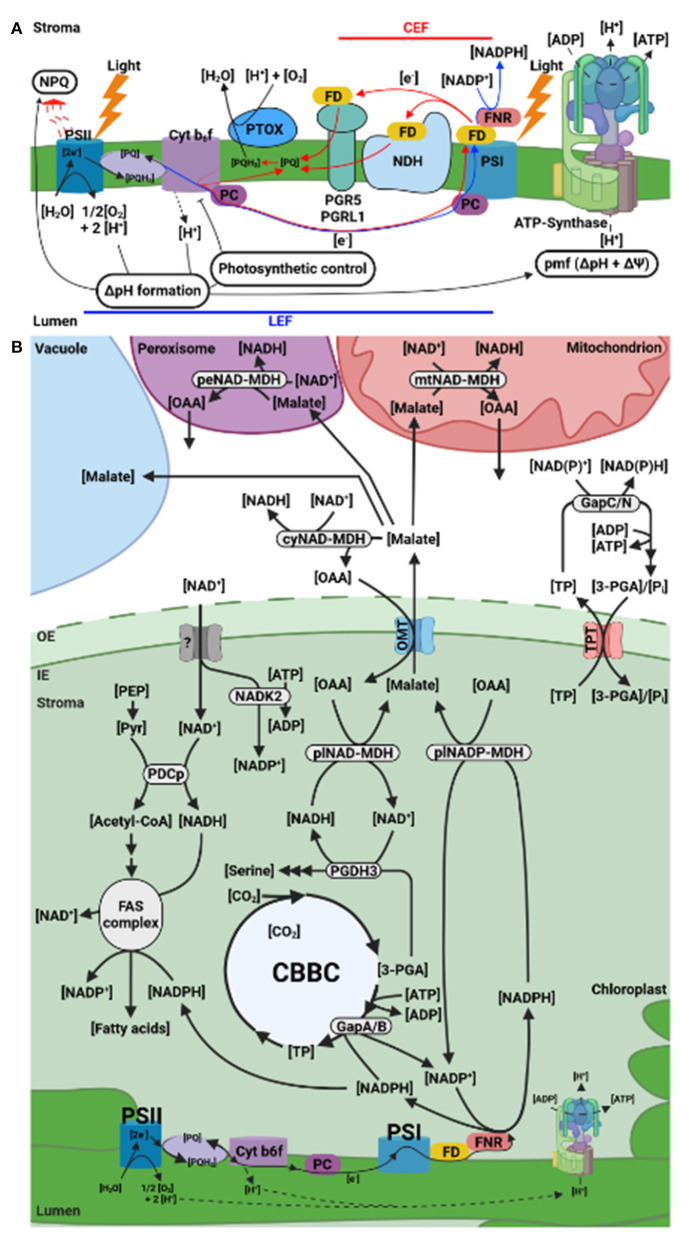
Plastid electron (e^−^) flow and indirect export of reducing equivalents. **(A)** Cyclic (red) and linear (blue) e^−^ flow/ transport (CEF and LEF, respectively) across the thylakoid membrane. Recycling e^−^ from reduced FD back to PQ by CEF allows for ATP production without NADPH formation. The pmf generated by LEF and CEF drives ATP synthesis at the ATP synthase complex. An excess of reduced PQH_2_ can be avoided via PTOX activity (modified from Yamamoto and Shikanai, 2020). **(B)** Indirect export of reducing equivalents from the stroma to prevent PSI acceptor side limitation. The terminal PSI e^−^ acceptor NADP is resupplied to FNR through NADPH oxidation in the CBBC and by export of surplus reducing equivalents out of the chloroplast. Fatty acid synthesis in the light may provide another e^−^ sink. Reducing power is transferred to the cytosol via TPT in form of TP or through OMT as malate. 3-PGA can be reduced to TP by GapAB in the plastid and be regenerated in the cytosol by GapC or GapN. Reduction of oxaloacetate to malate is driven by plNAD-MDH or plNADP-MDH fueled by the respective redox pools. The CBBC interconnects the NAD(H) and NADP(H) redox pools. This is mediated by PGDH3. Stromal NADP^+^ synthesis relies on NAD^+^ import and phosphorylation by NADK2. 3-PGA, 3-Phosphoglyceric acid; ADP, adenosine diphosphate; ATP, adenosine triphosphate; CEF, cyclic electron flow; cy, cytosolic; Cyt *b*_6_*f*, cytochrome *b*_6_*f*; FAS, fatty acid synthase; FD, ferredoxin; FNR, ferredoxin:NADP(H) oxidoreductase; GapA/B, bispecific (NAD^+^/NADP^+^-dependent) glyceraldehyde 3-phosphate dehydrogenase; GapC, cytosolic glyceraldehyde 3-phosphate dehydrogenase; GapN, non-phosphorylating irreversible glyceraldehyde 3-phosphate dehydrogenase; H, hydrogen; LEF, linear electron flow; MDH, malate dehydrogenase; mt, mitochondrial; NAD(H), nicotinamide adenine dinucleotide; NADK2, nicotinamide adenine dinucleotide kinase 2; NADP(H), nicotinamide adenine dinucleotide phosphate; NDH, NADH dehydrogenase; NPQ, non-photochemical quenching; O_2_, molecular oxygen; OAA, oxaloacetate; OMT, oxaloacetate malate transporter; PC, plastocyanin; PDCp, pyruvate dehydrogenase complex; pe, peroxisomal; PEP, 2-phosphoenolpyruvate; PGR5, proton gradient regulation 5; PGRL1, PGR5-like photosynthetic phenotype 1; Pi, inorganic phosphate; pl, plastid; pmf, proton motive force; PQ, plastoquinone; PQH_2_, plastoquinol; PSI, photosystem I; PSII, photosystem II; PTOX, plastid terminal oxidase; TP, triose-phosphate; TPT, triose-phosphate/phosphate translocator (Created with BioRender.com).

Recently, we reviewed the dynamic regulation of the proton motive force (pmf) across the chloroplast thylakoid membrane in response to light intensity shifts (Armbruster et al., 2017). In this mini review, we briefly revisit the electron transport in the photosynthetic machinery of C_3_ plants and the mechanisms that protect the two photosystems (PSII and PSI) at the level of the thylakoid membrane (Figure 1A). We then focus on the current understanding of the stromal redox pools downstream of PSI, plastid export of reducing equivalents, and new insights regarding the involved coenzymes (Figure 1B). We close by discussing future research directions.

## Electron Flow Between the Photosystems and Stromal Nadph Production

Light energy absorbed by the light harvesting complexes (LHC) causes charge separation in the chlorophyll a (Chl a) molecules located at the P680 reaction center of PSII (Figure 1A). Subsequently, the excited P680^*^ passes an electron (e^−^) on to the first e^−^ acceptor of the electron transport chain (ETC). The e^−^ gap in the oxidized P680^+^ molecule is filled through the splitting of water molecules which results in the release of e^−^ and molecular O_2_ (McEvoy and Brudvig, 2006). From P680^*^ e^−^ are shuttled via plastoquinone (PQ) to plastoquinol (PHQ) reduction through the lipid matrix of the thylakoid membrane to the cytochrome *b*_6_*f* complex (Cyt *b*_6_*f*). Cyt *b*_6_*f* transfers the e^−^ over to the luminal e^−^ carrier protein plastocyanin (PC). Reduced PC travels the longest distance in the ETC by diffusion to the PSI donor side where it re-reduces P700^+^ (Höhner et al., 2020). Through a second light-driven process an excited P700^*^ molecule is generated. At the PSI acceptor side, P700^*^ passes its e^−^ on to ferredoxin. Finalizing the linear e^−^ flow (LEF) reaction steps, the stromal enzyme ferredoxin-NADP^+^ oxidoreductase (FNR) reduces NADP^+^ to NADPH (Mulo, 2011). In summary, LEF transfers light energy captured by the thylakoid membrane-bound PS complexes into biochemically stored energy in the form of stromal NADPH. Together with ferredoxin, NADPH is the primary driver of stromal redox reactions and thus critical for the chloroplast redox poise (Geigenberger and Fernie, 2014). During LEF, the water splitting reaction and Cyt *b*_6_*f* activity release H^+^ into the lumen. This sets up a proton gradient ΔpH across thylakoid membrane. ΔpH and Δψ, the thylakoid membrane potential, together constitute the pmf, which drives stromal ATP production via ATP synthase (Kramer et al., 2003). NADPH and ATP fuel the Calvin Bassham Benson Cycle (CBBC) resulting in CO_2_-fixation, synthesis of triose-phosphates, and regeneration of NADP (Scheibe, 2004; Kramer and Evans, 2011; Dietz et al., 2016). NADPH consumption by the CBBC and other pathways relies on the dynamic growth conditions of plants (Gururani et al., 2015). A shortage of NADP ready to accept e^−^ results in PSI acceptor side limitation (Chaux et al., 2015; Kanazawa et al., 2017). In this situation O_2_ acts as an e^−^ acceptor triggering reactive oxygen species (ROS) production and concomitant PSI damage (Shimakawa and Miyake, 2018). Differently from PSII, which is repaired so rapidly that plants voluntarily sacrifice it if needed, PSI's turnover time is extremely long (Rantala et al., 2020). Consequently, plants have evolved several PSI protective mechanisms at the thylakoids and in the stroma including transport processes across the envelope membrane which effectively mitigate the risk of PSI damage (Munekage et al., 2002; Alric and Johnson, 2017).

## PSI Protective Mechanisms at the Thylakoid Membrane

A significant role in PSI protection can be assigned to the acidity of the thylakoid lumen. During light phases, low luminal pH activates the PsbS protein, which in combination with carotenoid pigments participating in the xanthophyll cycle regulates non-photochemical quenching (NPQ) (Jahns and Holzwarth, 2012). NPQ mostly causes heat dissipation at the PSII antenna which decreases the energy flux into the ETC and the amount of e^−^ rushing toward PSI via LEF. In addition, high ΔpH slows down PQH_2_ oxidation at Cyt *b*_6_*f* and therefore LEF e^−^ transport. This process is called photosynthetic control (Foyer et al., 2012).

Plant chloroplasts can also uncouple ATP from NADPH production in a process designated cyclic electron flow (CEF) around PSI. CEF diverts energy into the ATP pool without NADPH production (Shikanai and Yamamoto, 2017). This allows to finetune the ATP-NADPH stoichiometry according to the CBBC requirements and to decrease PSI acceptor side limitation by providing an alternative route for reduced ferredoxin (Kramer and Evans, 2011). Two CEF pathways exist in parallel: (1) PGR5/PGRL1 and (2) the NADH dehydrogenase-like (NDH) complex. Both utilize ferredoxin for PQH_2_ oxidoreduction. Their exact *in vivo* function and relative contribution to CEF are still under debate and very actively investigated (Shikanai and Yamamoto, 2017). An overaccumulation of reduced PQH_2_ can be prevented via the thylakoid membrane bound plastid terminal oxidase (PTOX). PTOX oxidizes PQH_2_ by reducing O_2_ to H_2_O (Alric and Johnson, 2017).

Lastly, imbalances between PSII and PSI excitation especially under fluctuating light conditions need to be avoided to prevent e^−^ excess at PSI (Grieco et al., 2012). Therefore, the mobile part of LHCII can switch between two states depending on reversible phosphorylation and either preferentially associate with PSII or PSI (state transition) (Pesaresi et al., 2011). LHCII phosphorylation in Arabidopsis is primarily catalyzed by the STN7 kinase. STN7 activity in turn, is partly coupled to the light dependent redox state of the PQ pool. Under low or ambient light conditions, the PQ pool remains oxidized. LHCII is docked to PSII and LEF prevails (State 1). Under light conditions that yield in a reduced PQ pool, i.e. buildup of PQH_2_ (such as fluctuating light), STN7 becomes active resulting in LHCII phosphorylation and migration to PSI (State 2). This situation supports CEF and overall protects PSI (Minagawa, 2011). Additionally, it was shown in Arabidopsis that acetylation of several photosynthesis components via the acetyltransferase NSI/ GNAT2 is also required for state transitions (Koskela et al., 2018, 2020).

## Mechanisms in the Stroma and at the Envelope Membrane That Indirectly Aid PSI Protection

Two export routes are known in C_3_ plant chloroplasts to indirectly provide reducing equivalents to other organelles to replenish stromal NADP (Figure 1B): the triose-phosphate/phosphate translocator (TPT) and the malate valve (aka malate-oxaloacetate (OAA) shuttle) (Dietz et al., 2016). Both contribute to the avoidance of PSI acceptor side limitation. Flux through the TPT is dependent on the prevailing Pi availability, which is coupled to the chloroplast's energy status, i.e., ATP availability (Flugge et al., 2011). The malate valve represents an independent alternative that connects chloroplast LEF with the cytosol, mitochondria, and peroxisomes via a process called malate circulation (Figure 1B) (Scheibe, 2004). Outside the plastid a plethora of dehydrogenases function to regenerated reducing equivalents in the respective organelles (Figure 1B). For detailed information on the characterization of these enzymes, we refer to several studies (Pracharoenwattana et al., 2007; Tomaz et al., 2010; Schneider et al., 2018; Liszka et al., 2020). The flux through the plastid malate valve will be the focus of the remaining article.

The plastid malate valve consists of an oxaloacetate/malate transporter (OMT1 or DiT1) in the inner envelope (Kinoshita et al., 2011) and two different malate dehydrogenases (MDH) to reduce stromal OAA to malate (Selinski and Scheibe, 2019). The two dehydrogenases differ in their coenzyme preference. The NADP-MDH is highly specific for NADP(H) (Scheibe, 1987), while the other isoform strongly prefers NAD(H) (NAD-MDH) (Berkemeyer et al., 1998). The co-existence of both MDHs has puzzled scientists for decades. The mainstream opinion has been that illuminated leaf chloroplasts use NAD merely to convert it into NADP via NAD kinase 2 (NADK2) (Figure 1B). NADH cannot be phosphorylated (Turner et al., 2004). Many reports have described FNR's strong coenzyme preference for NADP(H) (Correll et al., 1993; Piubelli et al., 2000; Medina et al., 2001; Hermoso et al., 2002). Therefore, photosynthesis and redox pathways should primarily depend on NADP(H) and not utilize NAD(H). It has also been assumed that transhydrogenase activity in the chloroplast stroma, i.e., enzymatic NADPH to NADH conversion, would affect photosynthesis by interfering with the carefully balanced NADP(H) production and consuming pathways, most importantly the CBBC (Krause and Heber, 1976). Until now, the vast majority of plastid biochemical pathways, especially the light-dependent and CO_2_ fixation reactions are depicted exclusively with NADP(H). However, if this were correct it seems hard to comprehend why C_3_ plant species possess NAD-dependent MDHs with confirmed enzyme activity in light and dark-incubated chloroplasts (Backhausen et al., 1998). Indeed, evidence suggesting a significant function for NAD(H) in illuminated chloroplasts has been growing over the last years (Zhao et al., 2018; Höhner et al., 2021).

## Diurnal Activity Pattern of the Two Different Plastid Malate Dehydrogenase Types

The NADP-MDH contains conserved cysteine residues in its N and C-terminus that function as a redox switch to keep the enzyme active only in the light, when the stroma is reduced (high availability of NADPH). *In vitro* this effect is achieved by adding DTT_red_ into extraction and assay buffers (Carr et al., 1999). Many experiments confirmed that NADP-MDH is highly active under reducing conditions but essentially inactive in the dark (Scheibe and Stitt, 1988). Surprisingly, several studies on chloroplast extracts from different C_3_ species reported substantial NAD-MDH activity regardless of the redox-state (Neuhaus et al., 1993; Backhausen et al., 1998; Selinski et al., 2014). This brought up the question: How can these two reactions coexist? One explanation is the different regulation of the two enzymes. NADP-MDH needs a reduced environment to function, while NAD-MDH isoforms, which lack several conserved cysteine residues, do not (Berkemeyer et al., 1998). Therefore, export of reducing equivalents in the light, especially in situations when the CBBC capacity and concomitant NADPH consumption drop, is facilitated via the NADP-MDH malate valve to ensure NADP for PSI (Okegawa and Motohashi, 2015; Thormählen et al., 2017; Selinski and Scheibe, 2019). However, at night NADP-MDH activity may create a futile cycle with the oxidative pentose phosphate pathway (OPPP) which produces NADPH (Kruger and von Schaewen, 2003; Sharkey and Weise, 2015). Therefore, NADP-MDH needs to stay inactive to avoid interference with the metabolism and risking plant survival during night phases.

Conversely, the physiologically assigned role for NAD-MDH has been participation in the chloroplast's dark metabolism, i.e., the incomplete glycolysis (Selinski et al., 2014). Under these conditions, stromal NADH level should be sufficiently high to drive the malate valve and decrease the reducing equivalent surplus while replenishing NAD for glycolysis. In other words, it was assumed that the absence of diurnal stromal NADH production would keep NAD-MDH inactive until dark metabolism takes over.

## Chloroplast MDH Isoforms—a Selection of Open Questions

While all these assumptions have merit, several discoveries from the C_3_ model plant Arabidopsis hint at a more complex scenario, suggesting NAD(H) may also play a role in daytime chloroplast metabolism.

1) Loss of the sole NADP-MDH gene does not result in a noticeable phenotype (Hebbelmann et al., 2012; Heyno et al., 2014). If C_3_ plants relied heavily on the NADP dependent malate valve, shouldn't we expect more dramatic effects especially on photosynthesis and PSI function in *NADP-mdh* loss-of-function lines? To our knowledge, PSI acceptor side limitation in *NADP-mdh* mutants has not been reported. Moreover, expression of an NADP-MDH variant that is no longer controlled by the redox poise and thus active during the night did not affect the mutants' growth under long-day conditions. This indicates that the enzyme activity did not significantly affect the OPPP as originally assumed. Interestingly, the mutants were more susceptible to fluctuating light and extended night periods (Yokochi et al., 2021).
2) If a lack of NADP-MDH could be backed up by NAD-MDH, as increases in *NAD-MDH* transcript level suggest (Selinski and Scheibe, 2014), what enzymes could aid in diverting e^−^ from NADPH into NADH so that it can function as a reducing agent to fuel the malate valve? Plants do not possess classic transhydrogenase enzymes as many bacteria do (Jackson, 2012).
3) By now we know a handful of NAD(H)-dependent stromal reactions. Is it really feasible that these strictly occur in the dark when according to the original hypothesis flux through plastid glycolysis would be sufficient to build up a decently sized stromal NADH pool? Disputing this idea, chloroplast fatty acid synthesis, which includes NAD(H)-dependent steps, is also active during the day (Browse et al., 1981).
4) What are the consequences of losing stromal NAD-MDH activity and the export of reducing equivalents via this route? How does it affect photosynthesis and PSI function? These questions have not been investigated but deserve research attention.

Recent findings on NAD-MDH mutants are highly intriguing but complicate future studies. Independent groups have shown that a homozygous *NAD-MDH* gene loss via T-DNA insertions is lethal (Beeler et al., 2014; Selinski et al., 2014). Also, a CRISPR/Cas9 approach to isolate loss of function NAD-*mdh* mutants was unsuccessful (Zhao et al., 2018). Posttranscriptional suppression of *NAD-MDH* mRNA via artificial microRNA resulted in severely underdeveloped and pale plant mutants (Beeler et al., 2014). Interestingly, *NAD-mdh* mutant lethality and visual phenotypes in T-DNA mutants were overcome by the expression of a non-functional NAD-MDH variant (Schreier et al., 2018). The authors showed that the developmental defects observed in *NAD-mdh* amiRNA lines are due to a second, non-enzymatic moonlighting function of the protein. Indeed, this and another study revealed protein-protein interactions between NAD-MDH and components of the heteromeric FtsH12-FtsHi AAA-ATPase complex (Kikuchi et al., 2018; Schreier et al., 2018). In theory, the non-functional enzyme complementing *NAD-mdh* line could serve as a stepping stone to dissect the NADH-dependent route via the malate valve. This could help to understand NAD-MDH's importance for the export of reducing equivalents and in replenishing NADP to PSI's acceptor side. However, these investigations are complicated because the lines were generated in a L*er*-1 (Landsberg *erecta*) background. Several studies on different Arabidopsis accessions have shown that small genetic variations play a big part in the phenotypic response to light regimes (Maloof et al., 2001; Van Rooijen et al., 2017; Kaiser et al., 2020). Thus, investigating the significance of either plastid MDH-type, which ideally involves the attempt to generate double mutants, is not straightforward. All publicly available *NADP-mdh* mutants are in Col-0 (Columbia) background.

A promising option is to employ the recently isolated viable *NAD-mdh* point mutant *som410* (*suppressor of mosaic death 1*). This non-functional NAD-MDH mutant resembles the wild type (Zhao et al., 2018). Interestingly, *som410* accumulates less biomass when grown in 16/8 h day-night cycles (long-day light conditions) but more biomass than wild-type plants if grown under constant light (Zhao et al., 2018). This apparent sensitivity to the length of the light period could imply a disturbed stromal redox poise and decreased photosynthesis. The authors suggest that plastid NADP-MDH and NAD-MDH can indeed simultaneously generate malate for export from chloroplasts during the day (Zhao et al., 2020). This would also mean that differently from the previous assumption transhydrogenase activity in the stroma does not occur during this process or it does not affect photosynthesis as originally postulated (Krause and Heber, 1976). The relative contribution of each respective MDHs to the malate valve flux remains to be shown. *som410* are Col-0 accession lines and thus may aid in resolving the flux question. This could further clarify the malate valve's importance for flawless PSI function.

## NADH Yielding Reactions in the Chloroplast Stroma

An alternative approach to shine light on the importance of the NAD-MDH driven malate valve and stromal NADH is to pinpoint the production routes of NADH in illuminated mesophyll chloroplasts. So far, three different plastid reactions are known to prefer NAD(H) over NADP(H): (1) NAD-specific glyceraldehyde-3-phosphate (Gap) dehydrogenases (*GapCp1* and *GapCp2*) which in Arabidopsis are expressed in heterotrophic tissues (Muñoz-Bertomeu et al., 2009). In chloroplasts, three additional Gap dehydrogenases (GapA1, GapA2, and GapB) exist. Interestingly, they possess dual co-enzyme specificity but only NAD(H)-dependent activity is light-independent (Baalmann et al., 1995; Berkemeyer et al., 1998; Sparla et al., 2002), (2) the plastid Pyruvate dehydrogenase complex (PDCp) which supplies NADH to the enoyl-acyl carrier protein (ACP) reductase (ENR) to initiate fatty acid synthesis (Camp and Randall, 1985), and (3) the phosphorylated pathway of serine biosynthesis (PPSB) (Ros et al., 2014).

PPSB is active in spinach leaves (Larsson and Albertsson, 1979) and therefore a good candidate for NADH production in illuminated chloroplasts. The Arabidopsis genome encodes three different Phosphoglycerate dehydrogenase (PGDH) isoforms (Benstein et al., 2013; Toujani et al., 2013). PGDH utilizes 3-PGA and NAD to catalyze the first of three steps to yield NADH and eventually serine in the stroma. Only *PGDH1* and *PGDH3* are expressed in leaves. Whereas PGDH3 is restricted to mesophyll cells, PGDH1 is found in autotrophic and heterotrophic tissues (Benstein et al., 2013; Toujani et al., 2013). Homozygous *pgdh1* loss of function mutants are not viable (Benstein et al., 2013). Interestingly, *PGDH1* and *PGDH3* loci differ greatly in their co-expression patterns: *PGDH1* clusters with *PGDH2* and other components of amino acid metabolism; PGDH3 is co-expressed with subunits of the thylakoid NDH complex suggesting a potential link to PSI and photosynthesis (Casatejada-Anchel et al., 2021; Höhner et al., 2021).

In part, these distinct features encouraged two independent studies focusing on the function of PGDH3 (Casatejada-Anchel et al., 2021; Höhner et al., 2021). The experiments showed that the PGDH3 isoform is a highly specific NAD(H)-dependent dehydrogenase, which is active in mesophyll chloroplasts under reducing conditions as found in the light (Höhner et al., 2021). In line with this, two independent *pgdh3* loss of function lines showed alteration in their photosynthetic performance. If grown under continuous long-day light conditions (16/8 h day-night cycles), *pgdh3* mutants revealed an increased transient NPQ level under non-saturating actinic light compared to wild-type plants (Höhner et al., 2021). This subtle behavior often indicates delayed CBBC activation and decreased ATPase NADPH consumption. Consequently, CO_2_ fixation in *pgdh3* mutants is lower than in wild-type plants (Casatejada-Anchel et al., 2021; Höhner et al., 2021).

Furthermore, *pgdh3* plants have increased PSI acceptor side limitations whereas photosynthetic control seemed unchanged. The metabolome of subcellular enriched fractions collected from *pgdh3* mutants suggested a shift in redox-related compounds. Stromal NADH level were lower, whereas the total NADPH pools increased. Interestingly, also the malate pools and thus malate circulation between organelles were subject to changes. Altogether the results point to a significant role of PGDH3 for C_3_ photosynthesis (Höhner et al., 2021). More importantly, the studies substantiate the evidence that stromal NADH is produced in mesophyll plastids during the day. Differently from PDCp, which works in tight conjunction with fatty acid metabolism, PGDH3 activity yields reduced NADH available for NAD-MDH and other stromal reactions. This provides a path to fuel the plastid malate valve with e^−^ originating from the stromal NADH pool. More importantly, because PGDH3 withdraws 3-PGA and its e^−^ from the CBBC, it represents an elegant indirect route to relay e^−^ from reduced NADPH into the NADH pool (Höhner et al., 2021). This aids in resupplying oxidized NADP to the PSI acceptor side. The involvement of the tightly regulated CBBC allows the chloroplast to keep close tabs on the pathway's flux thereby avoiding the risk of uncontrolled transhydrogenase activity in the stroma that may affect photosynthesis (Höhner et al., 2021).

## Physiological Conditions That Require Increased Stromal NADH Production and Indirect Export of Reducing Equivalents From the Chloroplast

Arabidopsis upregulates *PGDH3* transcription and protein level if plants are subjected to dynamic light stress (Schneider et al., 2019; Niedermaier et al., 2020). Indeed, *pgdh3* loss-of-function mutants revealed progressing PSII damage (low *F*_v_/*F*_m_) when fluctuating growth light was applied. In contrast, *pgdh3* plants were capable to adjust to constant highlight stress which suggests other mechanisms provide protection under such conditions. Altogether, the data indicate that the NADH-dependent pathway provides a well-needed alternative route to export reducing equivalents from the stroma (Höhner et al., 2021). This is critical during growth phases with sudden light intensity changes, which occur frequently in the field. Since PSI damage occurs when e^−^ acceptors become limited (Miyake, 2020) it is likely that the lack of PGDH3 and decreased stromal NADH production also result in PSI damage. This needs to be tested in the future as it may explain why gene expression of *PGDH3* and components of the NDH complex appears to be coordinated (Höhner et al., 2021). Although the structure suggests reduced ferredoxin is the electron donor to NDH (Laughlin et al., 2019; Schuller et al., 2019), *in vitro* experiments have successfully employed NADH and NADPH as the primary e^−^ donors to drive NDH activity probably *via* FNR (Sazanov et al., 1998; Rumeau et al., 2005; Strand et al., 2016, 2017). Overall, a coordinated increase in NDH and PGDH3 activity in response to stromal NADPH buildup may be necessary to minimize the risks of PSI damage.

## Further Research Directions Toward Understanding the Importance of Diurnal Stromal NAD(H)

A full understanding of the stromal NAD(H)-dependent pathway in illuminated chloroplasts could inspire new strategies to secure plant productivity during light stress e.g., by protecting PSI through avoiding acceptor side limitations and concomitant damage to PSI. To reach this goal we suggest a few research directions and potential challenges.

### On Stromal NADH Production

Quantitative information of the alternative NADH yielding reactions is required. Since substantial amounts of stromal NADH remain in *pgdh3* mutants (Höhner et al., 2021), it will be important to pinpoint how much if any is contributed by PGDH1 activity in mesophyll chloroplasts (PGDH2 is not expressed). Since PGDH1 loss of function is lethal (Höhner et al., 2021) this requires a knock-down approach in the *pgdh3* background. In parallel, it needs to be determined to what degree PDCp generates a reduced coenzyme surplus, i.e. NADH unused in diurnal fatty acid synthesis available to drive the malate valve. GAPCp1 and GAPC2 are reportedly not expressed in Arabidopsis leaves (Muñoz-Bertomeu et al., 2009). This should be validated in wild-type and *pgdh* mutants to ensure no compensatory upregulation of the enzymes take place if stromal NADH production routes are blocked.

Regulatory aspects could also be further explored for PGDH enzymes. For instance, all three PGDHs were shown to bind thioredoxin (TRX). However, only PGDH1 seemed to be directly regulated by TRX according to *in vitro* studies (Yoshida et al., 2020). Additionally, product or feedback inhibition by serine was shown for PGDH1 and PGDH3 isoforms from Arabidopsis (Benstein et al., 2013; Okamura and Hirai, 2017). Protein-protein interactions between PGDH proteins and unknown binding partners may exert another level of regulation. PGDH1 and PGDH3 display distinct localization in leaf plastids. Although a strong UBQ10 promoter from Arabidopsis was used in these studies, PGDH1 appeared soluble in the stroma whereas PGDH3 remained in small punctured structures in the chloroplasts of Arabidopsis and tobacco (Höhner et al., 2021). Endogenous promoters driving expression of PGDH fluorescence protein fusions and immunoblotting of plastid sub-organellar fractions should settle this discussion. Lastly, a moonlighting function for PGDH3 cannot be excluded at this point.

### On the Activity and Importance of the NAD(H)-Dependent Route to Drive the Malate Valve

It will be critical to further exploit assays that allow activity monitoring of either MDH type simultaneously. This could be realized through employing genetically encoded sensors. A number of studies from Arabidopsis report significant progress toward this goal (Okamura and Hirai, 2017; Lim et al., 2020; Steinbeck et al., 2020). Here, NAD(H) and NADP(H)-specific sensors were targeted to the cytosol, stroma, and mitochondria. The data not only confirmed a light-dependent redox shunt from the stroma into the cytosol but also showed clear evidence for stromal NADH reduction in response to light (Lim et al., 2020; Steinbeck et al., 2020). In the future, these measurements need to be realized in a single reporter line that expresses NAD(H) and NADP(H) sensors in the same organelle. Introgressing these sensors into loss and gain-of-function mutants of interest will allow to determine the relative contribution of specific enzymes, transporters etc.

A first step into this direction was realized through the design of an illumination platform synchronized imaging setup (Elsässer et al., 2020). Elsässer and colleagues managed to trigger dark-light transitions similar to conditions expected in the field while almost simultaneously recording cytosolic NAD(H), stromal ATP, and stromal pH changes via a genetically encoded sensors in wild-type and *NADP-mdh* Arabidopsis mutants. The experiments on true leaves documented a strong light-dependent NADH reduction in the wild type but also *NADP-mdh* mutant cytosol. They also found that dark, i.e., resting, cytosolic NADH/NAD ratios were higher in *NADP-mdh* than in wild-type plants. The authors concluded that a significant export of reducing equivalents from illuminated chloroplasts persists in the absence of stromal NADP-MDH. Interestingly, carbon and reductant fluxes between the organelles remain high during the night, which was underappreciated thus far. Altogether, these findings may indicate a more important role of the stromal NAD-dependent MDH flux via the chloroplast malate valve and the reactions that deliver reduced NADH then previously acknowledged.

### On the Characterization of Coenzyme Specificity for (Plastid) Dehydrogenases

Finally, we propose that characterization of (stromal) NAD(P) dehydrogenases should generally determine V_max_, K_m_, K_cat_ etc. for both coenzymes, NAD(H) and NADP(H). When we started our research, we were surprised how little information was available in the newer literature. Over 30 years ago, a dual coenzyme specificity for NAD(H) and NADP(H) was shown for the chloroplast GapA/B isoforms. Other NAD(H)-dependent reactions or enzymes with dual coenzyme specificity may have been overlooked. Regardless if additional NAD(H) or dual coenzyme specific dehydrogenases will emerge, the data will greatly improve our perspective of the stromal redox poise.

## Conclusions

Understanding the relative light-dependent activity of NADP(H) vs. NAD(H)-coupled reactions in the stroma and their contribution to malate export from the chloroplast will yield a better understanding of C_3_ photosynthesis and the constraints their flux has on photosynthetic efficiency. Once this has been achieved, engineering these pathways could improve plant yields in the field, for instance through avoidance of PSI acceptor side limitations and irreversible PSI damage. Even decades after their first description, the chloroplast malate valve and its two MDH enzymes remain therefore highly interesting. Thorough future research employing a combination of new and more traditional tools such as *in vivo* imaging of genetically encoded sensors and chlorophyll fluorescence spectroscopy of plants exposed to standard and dynamic growth conditions may yield insights to unlock the full potential of the malate valve and its linked pathways for biotechnological applications.

## Author Contributions

H-HK wrote the manuscript. MK edited text, references, and designed the figure. Both authors contributed to the article and approved the submitted version.

## Funding

H-HK was funded by the 3rd call ERA-CAPS grant (NSF IOS-1847382) and a National Science Foundation Career Award (IOS-1553506).

## Conflict of Interest

The authors declare that the research was conducted in the absence of any commercial or financial relationships that could be construed as a potential conflict of interest.

## Publisher's Note

All claims expressed in this article are solely those of the authors and do not necessarily represent those of their affiliated organizations, or those of the publisher, the editors and the reviewers. Any product that may be evaluated in this article, or claim that may be made by its manufacturer, is not guaranteed or endorsed by the publisher.
